# The relationship of the male's proctodeal gland size to sperm-egg interaction and the duration of fertility in Japanese quail

**DOI:** 10.1016/j.psj.2024.103809

**Published:** 2024-05-03

**Authors:** Ahmed Abdel-Kareem Abuoghaba, Mostafa Galal Abdelfattah, Mohamed Abdelhamid Mohamed Sayed, Mohamed Hosny

**Affiliations:** ⁎ Sohag University Faculty of Agriculture, Sohag, Egypt; † Assiut University Faculty of Agriculture, Assiut, Egypt; ‡ Al-Azhar University Faculty of Agriculture, Assiut Branch, Assiut, Egypt

**Keywords:** proctodeal gland, Japanese quail, sperm penetration, fertility duration, testosterone

## Abstract

In avian species, male fertility significantly impacts reproductive success. This study investigates the relationship between proctodeal gland size in male Japanese quails and sperm function, as well as female fertility duration. Six hundred adult Japanese quails were selected and housed in individual cages. Males (n = 300) were divided into 6 groups (50/group) based on the size of their proctodeal glands. Females (n = 300) were randomly assigned to corresponding groups. After acclimatization, sperm kinematics and the frequency and weight of foam discharge were evaluated. Males were paired with females for 24 h, and eggs were collected for 20 d postcopulation. Eggs were incubated to assess fertility duration. Fresh eggs (n = 20/group/d) were used to assess sperm penetration into the perivitelline membrane on the 2nd, 9th, and 16th d postinsemination. Plasma testosterone levels and the testes' relative weight were determined. The results indicated a significant increase (*p* < 0.0001) in sperm concentration, total and progressive motility, and nearly all sperm kinematic parameters such as VSL, VCL, VAP, LIN, WOB, and STR values as the size of the proctodeal gland increased. Quails copulated with males having a small and average proctodeal gland area (<400 mm^2^) laid fertile eggs for a shorter period and had significantly fewer sperm penetration holes than those mated with males having a larger proctodeal gland area (>400 mm^2^). The proctodeal gland size was positively correlated with testicular weight, plasma testosterone concentrations, and sperm kinetic variables. The results indicate that the size of the proctodeal gland in males can be used to predict sperm function and the duration of fertility in Japanese quail.

## INTRODUCTION

In sexually mature male Japanese quail, measuring the proctodeal gland size and the amount of foam discharge is used to predict testicular function and consequently sperm fertilizing capacity (Biswas et al., 2007a,b). The development of the proctodeal gland and its foam production depend on androgen concentrations and the physiological state of the testes. The size of the proctodeal gland is positively correlated with the testes size, sexual activity, plasma testosterone concentration, and thus fertilization ability in sexually active male quail (Biswas et al., 2007a,b and Singh et al. 2011 a,b).

Male Japanese quail secrete foam from their proctodeal gland, which is introduced into the female reproductive tract during copulation. This foam enhances the survival and activity of sperm when mixed with semen, thus improving sperm motility (Singh et al., 2011b; Farooq et al., 2015). It suspends sperm in the female's proctodeum to prevent sperm loss during oviposition and enables the slow release of sperm from the sperm storage site, thereby improving fertilization (Cheng et al., 1989). Additionally, the foam helps fill sperm storage tubules, contributing to maintaining a high fertilization rate (Finseth et al., 2013). It consists of a viscous glycomucoprotein, which includes sulfur and non-sulfur mucopolysaccharides. This composition forms when proctodeal bacteria, such as *Escherichia coli and Proteus mirabilis*, metabolize glucose in the secreted mucosa, producing CO_2_ and H_2_. These bacteria may utilize the sugars secreted by the foam gland and convert them into lactate (Mohan et al., 2004). High levels of lactate in the foam and lactate dehydrogenase in seminal plasma can serve as an energy source, enhancing sperm metabolic activity and maintaining sperm motility in the oviduct for a longer time after copulation.

Under in vitro conditions, sperm form clusters when foam is absent in the semen. However, when the foam is added to the semen, the sperm disaggregate, maintain normal morphology, remain motile for an extended time, and show higher metabolic activity (Fujihara and Koga, 1991; Singh et al., 2011b; Singh et al., 2012). It is preferable to select males with large-sized proctodeal glands to achieve higher fertility (Mohan et al., 2002; Biswas et al., 2007a, b).

The correlation between the size of the proctodeal gland, sperm motility, kinetic variables, foam discharge, and testosterone concentrations in Japanese quail is well documented in the literature. However, available data regarding a relationship that may exist between the proctodeal gland size, sperm-egg interaction, and fertility duration is lacking. Therefore, this study aims to investigate the relationship between the proctodeal gland size, sperm fertilizing capacity, and fertility period in Japanese quail.

## MATERIALS AND METHODS

The experiment was carried out at the Poultry Research Farm, Department of Poultry Production, Faculty of Agriculture, Assiut University, Assiut, Egypt. All procedures of the current study were conducted following the University guidelines for the care of experimental animals. Ethical approval (07-23-04) was obtained from the Research Ethics Committee of the Faculty of Agriculture, Assiut University, Egypt. The study was reported following ARRIVE guidelines.

### Experimental Design

This study utilized 600 eight-wk-old male and female Japanese quail (*Coturnix japonica*). The males (n = 300) were divided into 6 groups, each consisting of 50 males, based on their proctodeal gland area (**PAREA**). Similarly, the females (n = 300) were randomly assigned to 6 groups, with 50 females in each group. The proctodeal gland area was measured according to Siopes and Wilson (1975) and Chaturvedi et al. (1993). The measurements of the proctodeal area were ≤250, 251–300 (small), 301–350, 351–400 (average), 401–450, and > 450 mm^2^ (large) for groups from 1 to 6, respectively. The classified males according to their proctodeal area (gland size) were housed in individual wire cages (20 × 20 × 25 cm) under uniform husbandry conditions. Females (n = 300) were also randomly divided into 6 groups and housed in individual cages. The birds were exposed to an ambient temperature ranged from 22 to 26°C, relative humidity of 50–60%, and a photoperiod of 14/10 h light/dark cycle, provided with fresh water and offered feed ad libitum. The birds were left 2 wk to acclimatize and fed on a quail breeder diet containing 20% crude protein and 3,000 kcal/kg metabolizable energy.

### Determination of Proctodeal Gland Measurements

The lateral width and dorsal-ventral height of the proctodeal gland of each male were measured to the nearest 0.01 mm using Vernier digital calipers to calculate the PAREA. PAREA was used as an index of gland size and was calculated according to the formula: PAREA (mm^2^) = (proctodeal gland height × width) as proposed by Siopes and Wilson (1975) and Biswas et al., (2007a,b). The proctodeal gland volume (PVOL) was calculated according to the formula: 4/3 × 3.5414 × a × b^2^, where a = 0.5 × long axis and b = 0.5 × short axis (Chaturvedi et al., 1993; Satterlee et al., 2002).

### Quantitative Measurement of Foam Production

The determination of natural foam secretion involved counting the frequency of foam release over 24 h by observing the number of foam masses in the fecal trays under each bird. Following each count of foam discharge, the trays were promptly cleaned and replaced in the cages for the subsequent count. Additionally, quantitative measurement of foam production was conducted by collecting the foam produced by gently squeezing the proctodeal gland of each male. The foam produced by each bird was collected in separate airtight glass bottles to prevent evaporation and weighed immediately using an electronic analytical balance with precision up to 4 decimal places (Mohan et al., 1991).

### Semen Collection and Semen Quality Evaluation

Semen collections were performed at 10 and 11 wk of age. The semen was collected from 10 birds/group using the abdominal massage technique described by Burrows and Quinn (1937). Special care was taken to avoid contamination by proctodeal gland foam, feces, and watery fluids from the proctodeal region. This was accomplished by clipping the feathers around the vent region and by cleaning the proctodeal gland gently with tissue paper before collection. The male was gently restrained in the palm of the left hand and proctodeal foam was collected separately in aluminum paper by delicately squeezing the lateral wall of the cloaca just before semen collection and the foam was weighed after semen collection. Semen was collected after the lumber region toward the tail was massaged 3 to 4 times smoothly and gentle pressure was applied on either side of the vent using the thumb and forefinger as described by Gavora and Hodgson (1976) and Burrows and Quinn (1937). The semen was collected in graduated capillary tubes to quantify the ejaculate volume (Hanafy and Khalil, 2015).

### Sperm Kinematic Parameters

After semen collection, sperm kinematics were assessed using computer assisted sperm analysis CASA (Mira-9000, Sperm Analyzer CASA software, Mira Lab, Egypt). Before each assessment, the system was calibrated for quail sperm morphometric properties. Samples (n = 10/group) were first diluted at 1:7 with phosphate-buffered saline (**PBS**) prior to measuring. To record motility, 5 µL drop of diluted semen was loaded onto a pre-warmed CASA slide chamber (10 μm, 4-chamber slide, Leja Products B.V., Nieuw-Vennep, Netherlands) that was placed on a heated stage (37ºC). To prepare the samples for recording sperm motility, 45 s were allowed to elapse (i.e., 30 s equilibration time before loading, and 15 s settling time after sample loading on the slide). For dynamic sperm analyses, the slides were covered with a 10 × 10 mm coverslip. For each sample, at least 200 tracks of motile spermatozoa from a minimum of 10 random fields were recorded using Spermolyzer software (Spermolyzer, Mira Lab, Egypt) using a phase-contrast microscope (Leica Microsystems GmbH, Wetzlar, Germany) at 40 × magnification for sperm motility and velocity variables. Spermatozoa with average path velocity (**VAP**) < 10 µm/s and straight-line velocity (**VSL**) < 5 µm/s are considered immotile. The CASA defined thresholds were 20 VAP and 80% straightness (STR). The percentage of spermatozoa demonstrating progressive motility were calculated as the number of spermatozoa exceeding 20 µm/s VAP and 80% STR divided by the number of motile spermatozoa (Sayed et al., 2017). Fields were randomly captured to eliminate bias toward higher or lower sperm concentration or motility areas. Fields that included debris or clumps of sperm were excluded from the analysis. The following sperm motility parameters were determined; sperm total motility percentage (**TMOT** %), nonprogressive motility (NP %; all other patterns of motility with an absence of progression), immotility (IM %), straight linear velocity (VSL, μm/sec), curvilinear velocity (VCL, μm/sec), average path velocity (VAP, μm/sec), linearity (LIN % = VSL/VCL), wobble movement coefficient (WOB % = VAP/VCL), straightness (STR % = VSL/VAP), mean angular degree (MAD, º), amplitude of lateral head displacement (ALH, μm), and beat-cross frequency (BCF, Hz).

### Determination of Fertility Duration and Sperm Penetration

At 12 wk of age, males from different groups (n = 300) were transported to their corresponding female cages (n = 300) for 24 h and then removed. Eggs were collected for 20 d postinsemination. Forty eggs/group/day (except on the 2nd, 9th, and 16th d postcopulation, where 20 eggs/group/d were incubated) were selected and put into labeled pedigree boxes and incubated to determine the fertility duration. The incubator temperature was set at 37.5°C and a relative humidity of 60 to 65% was maintained. The eggs were incubated for 1 wk, then cracked to identify any embryonic growth and calculate fertility. The fertility percentage was calculated by dividing the number of fertile eggs by the number of total eggs set in the incubator multiplied by 100.

On the 2nd, 9th, and 16th d postinsemination, fresh eggs (n = 20/treatment group/d) were used to count the holes in the perivitelline membrane overlying the germinal disc (area = 9 mm^2^) hydrolyzed by sperm. A portion (approximately 9 mm^2^) of the ovum perivitelline layer was removed, rinsed in saline, straightened on a microscope slide, fixed with 20% formalin, and stained with the Schiff's fuchsin-sulfite reagent (Sigma-Aldrich Co., St. Louis, MO). A light microscope was used to count the holes caused by sperm penetration in the stained portion of the perivitelline membrane (Bramwell et al., 1995; Bramwell and Howarth, 1997).

### Libido Time

Libido time was estimated from the time that the male was placed inside the female's cage to the point when the male started to mount the female.

### Blood Collection and Testicular Measurements

At the end of the experiment, blood samples from twenty randomly chosen males per treatment were collected from jugular vein in heparinized tubes, centrifuged at 3,000 rpm for 15 min to separate plasma and were stored at −20°C until needed. The randomly selected males were sacrificed, and the left and right testes were removed and weighed to calculate the (testis’ relative weight = testicles weight/body weight × 100). The right and left testis length and width were measured using a sensitive digital caliper (150 mm with 0.02 mm precision). The right and left testis shape index was obtained by dividing the testis width by the testis length.

### Estimation of Testosterone Concentration

Testosterone concentrations were assayed in plasma using a testosterone enzyme immunoassay test kit (**ELISA**) procured from Biocheck, Inc. (Foster City, CA). The absorbance was measured within 15 min using a microplate reader (BioTek ELX 808 IU, Agilent Technologies, Inc., Santa Clara, CA) set at a wavelength of 450 nm.

## STATISTICAL ANALYSIS

The obtained data were analyzed by analysis of variance ANOVA using the GLM procedure of SAS (SAS Institute, 2009). When treatment effects were significant, differences between least squares means were tested using Duncan's multiple-range test and the differences were considered significant at the level of *P* < 0.05 (Duncan,1955). Before analysis, data normality was assessed using the Shapiro-Wilk test (considered normal if *P* > 0.05). Non-parametric data were subsequently analyzed using the Kruskal-Wallis test, followed by post-hoc analysis using the Dunns pairwise multiple comparison test with Bonferroni correction. Statistical analyses were conducted using IBM SPSS Statistics 24.0 software (IBM Deutschland GmbH, Ehningen, Germany). Pearson correlation coefficient for some parameters was also performed (Steel and Torrie, 1980).

## RESULTS

The mean values of PAREA, PVOL, foam weight, foam discharge frequency, libido time, combined testes weight, testes’ relative weight (%), and testosterone concentrations in different groups of Japanese quails are presented in Table 1.

**Table 1:** The mean values of body weight, proctodeal gland area, volume, foam weight, foam discharge frequency, libido time (in 50 males/group), and combined testes weight, gonadosomatic index (%), and plasma testosterone concentrations (in 20 randomly selected males/group) in different groups of Japanese quails.

Parameters	Range of proctodeal gland index (mm^2^)						Pooled SEM	*p* value
	1st G (≤250)	2nd G (251-300)	3rd G (301-350)	4th G (351-400)	5th G (401-450)	6th G (>450)		
Body weight (g)	206.0^e^	217.7^d^	221.2^cd^	229.7^b^	226.6^bc^	239.1^a^	2.67	0.0001
PAREA (mm^2^)	229^f^	276^e^	322^d^	376^c^	426^b^	503^a^	2.87	0.0001
PVOL (mm^3^)	1893^f^	2527^e^	3220^d^	4062^c^	4926^b^	6293^a^	58.53	0.0001
FW (mg/bird)	18.4^e^	46.6^d^	53.3^d^	78.7^c^	100.3^b^	123.71^a^	5.38	0.0001
Frequency of foam discharge (24 h/bird)	14.5^c^	16.2^c^	18.9^bc^	18.7^bc^	22.9^ab^	27.8^a^	1.74	0.0002
Libido time (s)	21.5^a^	14.8^b^	15.9^b^	8.7^c^	3.0^d^	2.7^d^	1.93	0.0001
Testosterone (ng mL^−1^)	0.90^c^	1.11^c^	1.59^bc^	1.54^bc^	2.38^ab^	2.91^a^	0.32	0.0004
Combined testes weight (g)	5.51^c^	5.65^c^	7.30^b^	7.69^b^	8.39^ab^	9.33^a^	0.45	<.0001
Relative testis weight (%)	2.65^d^	2.61^d^	2.82^cd^	3.17^bc^	3.59^b^	4.32^a^	0.16	<.0001

a,b Means with at least one common superscript in a row do not differ significantly (*p* > 0.05). PAREA (mm^2^) = Proctodeal gland area, PVOL (mm^3^) = proctodeal gland volume, FW (mg/bird) = Foam weight. * *p* < 0.05; NS = *p* > 0.05.

The mean values of cloaca area (PAREA) in groups 1 to 6 were in ascending order, with the means averaged 229.50 mm^2^ in group 1 and increased linearly to 503.84 mm^2^ in group 6. The mean values of cloaca volume (PVOL) followed the same pattern as PAREA. The volume of foam discharge was higher in males in group 6 compared to those in groups 1, 2, 3, and 4. Group 5 had a foam discharge count that was intermediate between group 6 and groups 3 and 4. This was reflected in the foam weight, which was significantly higher in group 6 compared to the rest of the groups. Males in group 6 had significantly heavier combined testes weights than those in groups 1, 2, 3, and 4. The testes’ relative weight in group 6 was significantly (*P* < 0.05) higher compared to all other groups. Significant differences in plasma testosterone concentrations and libido time were observed among different groups. Males with PAREA > 450 mm^2^ (group 6) had higher plasma testosterone concentrations than those with a PAREA < 400 mm^2^ (groups 1, 2, 3, and 4). The results also showed that males from groups 5 and 6 took a shorter time to copulate with females (libido time).

Table 2 shows ejaculate volume, sperm concentration and sperm kinematic parameters in ejaculates of different groups. It was observed that sperm concentration, sperm total and progressive motility and almost all sperm kinematic parameters, i.e., VSL, VCL, VAP, LIN, WOB, and STR values, were (*P* < 0.0001) increased as proctodeal gland size increased.

**Table 2:** Ejaculate volume, sperm concentration and sperm kinematic parameters in ejaculates (n = 10/group) of different quail groups.

Parameters	Range of proctodeal gland index (mm^2^)						SEM	*p* value
	1st G (≤250)	2nd G (251-300)	3rd G (301-350)	4th G (351-400)	5th G (401-450)	6th G (>450)		
Ejaculate volume (μL)	9.5^c^	10.1b^c^	11.8^b^	15.7^a^	15.7^a^	17.6^a^	0.72	0.0001
Sperm concentration (*10^6^/mm^3^)	430.6^f^	496.0^e^	598.8^d^	639.7^c^	719.9^b^	760.5^a^	11.44	<.0001
TMOT (%)	88.8^c^	90.9^bc^	92.9^b^	95.7^a^	96.4^a^	97.1^a^	0.77	<.0001
PR (%)	36.4^d^	44.7^c^	45.4^c^	51.7^b^	56.3^ab^	58.5^a^	1.704	<.0001
IM (%)	11.2^a^	9.1^ab^	7.1^bc^	5.3^cd^	3.6^d^	2.9^d^	0.85	<.0001
VSL (μm/s)	35.7^e^	37.5^de^	40.4^d^	47.5^c^	57.0^b^	63.9^a^	1.041	<.0001
VCL (μm/s)	118.3^c^	106.3^d^	112.2^cd^	119.5^c^	134.4^b^	145.3^a^	3.43	<.0001
VAP (μm/s)	76.2^bc^	62.5^d^	67.4^cd^	76.2^bc^	86.2^ab^	94.9^a^	4.34	<.0001
LIN (%)	28.5^d^	32.7^c^	34.2^c^	38.7^b^	42.4^a^	44.1^a^	1.09	<.0001
WOB	54.3^e^	57.0^d^	59.5^c^	63.4^b^	64.9^ab^	65.9^a^	0.83	<.0001
STR (%)	49.7^d^	55.7^c^	54.5^c^	60.6^b^	64.4^a^	66.4^a^	1.289	<.0001
MAD	95.6^a^	86.8^bc^	87.7^b^	79.0^c^	82.3^bc^	83.3^bc^	2.58	0.0003
ALH (μm)	11.9^a^	11.4^ab^	11.2^ab^	10.0^c^	10.7^bc^	10.9^bc^	0.30	0.0005
BCF (Hz)	3.4^a^	2.9^b^	2.7^b^	2.0^c^	2.2^c^	2.2^c^	0.11	<.0001

a–d Means with at least one common superscript in a row do not differ significantly (*p* > 0.05). Each value represents the mean ± standard error of the mean of 40 ejaculates. Abbreviations: TMOT, sperm total motility percentage; PR, progressive motility (%); IM, immotile sperm (%); VSL, velocity straight line; VCL , velocity curvilinear; VAP , velocity average path; LIN , linearity; WOB, wobble movement coefficient; STR , straightness; MAD, mean angular degree; ALH , lateral head displacement; BCF , beat-cross frequency. *p* < 0.05, NS = *p* > 0.05.

Males in groups 4, 5, and 6 returned larger ejaculate volumes compared to those in groups 1, 2, and 3. Generally, males with larger proctodeal glands had higher sperm motility (*P* < 0.05) and velocity values compared to those with small sized glands. Sperm in ejaculates from quails with proctodeal gland sizes above 450 mm^2^ showed higher VCL, VSL, and VAP values than those in ejaculates from other groups. Males in groups 5 and 6 had comparable total and progressive motility, percentages of immotile sperm, LIN, STR, and BCF, which differed significantly from those in groups 1, 2, and 3 (males with proctodeal gland sizes < 351 mm^2^).

In addition, sperm from ejaculates of birds with proctodeal gland sizes < 250 mm^2^ exhibited higher values of MAD, ALH, and BCF than those in the ejaculates of males in other groups.

**Figure 1:**
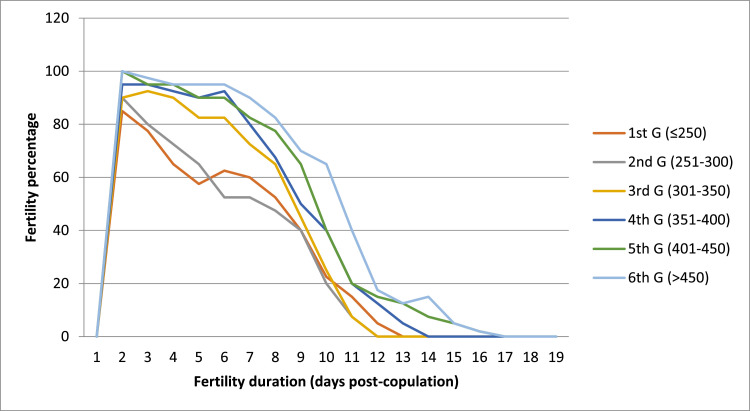
The percentages of fertile eggs and the number of days that quails in the different groups laid fertile eggs postcopulation.

The percentages of fertility and the number of days that quails in the different groups laid fertile eggs postcopulation are shown in Table 3 and Figure 1.

**Table 3:** The percentages of fertile eggs and the number of days that quails in the different groups laid fertile eggs postcopulation.

Fertility period	Range of proctodeal gland index (mm^2^)							*p-* value
(days)	1st G (≤250)	2nd G (251-300)	3rd G (301-350)	4th G (351-400)	5th G (401-450)	6th G (>450)	SEM	
1	-	-	-	-	-	-	-	-
2	85.00^d^	90.00^c^	90.00^c^	95.00^b^	100.00^a^	100.00^a^	0.66	<.0001
3	75.00^e^	80.00^d^	92.50^c^	95.00^b^	95.00^b^	97.50^a^	0.69	<.0001
4	65.00^e^	72.50^d^	90.00^c^	92.50^b^	95.00^a^	95.00^a^	0.72	<.0001
5	57.50^e^	65.00^d^	82.50^c^	90.00b	90.00^b^	95.00^a^	0.61	<.0001
6	52.50^e^	52.50^e^	82.50^d^	92.50b	90.00^c^	95.00^a^	0.54	<.0001
7	60.00^e^	52.50^f^	72.50^d^	80.00^c^	82.50^b^	90.00^a^	0.66	<.0001
8	52.50^e^	47.50^f^	65.00^d^	67.50^c^	77.50^b^	82.50^a^	0.74	<.0001
9	40.00^e^	40.00^e^	45.00^d^	50.00^c^	65.00^b^	70.00^a^	0.63	<.0001
10	22.50^d^	20.00^e^	25.00^c^	40.00^b^	40.00^b^	65.00^a^	0.80	<.0001
11	15.00^c^	7.50^d^	7.50^d^	20.00^b^	20.00^b^	40.00^a^	0.31	<.0001
12	5.00^d^	0.00^e^	0.00^e^	12.50^c^	15.00^b^	17.50^a^	0.28	<.0001
13	0.00^c^	0.00^c^	0.00^c^	5.00^b^	12.50^a^	12.50^a^	0.26	<.0001
14	0.00^c^	0.00^c^	0.00^c^	0.00^c^	7.50^b^	15.00^a^	0.11	<.0001
15	0.00^b^	0.00^b^	0.00^b^	0.00^b^	5.00^a^	5.00^a^	0.13	<.0001
16	0.00^b^	0.00^b^	0.00^b^	0.00^b^	2.00^a^	2.00^a^	0.13	<.0001
17	0.00	0.00	0.00	0.00	0.00	0.00	-	-
18	0.00	0.00	0.00	0.00	0.00	0.00	-	-
19	0.00	0.00	0.00	0.00	0.00	0.00	-	-

a–d Means with at least one common superscript in a row do not differ significantly (*p* > 0.05). Forty selected eggs /group/day were used to determine fertility percentage.

The data showed that females copulated with small (<301 mm^2^), and average-size (301–400 mm^2^) proctodeal gland males produced fertile eggs for 12 and 13 d postinsemination, respectively. While females mated with large-size gland males (>400 mm^2^) continued producing fertile eggs for 16 d. In addition, fertility percentages in large-size gland groups were higher than in small and average-size gland groups.

**Figure 2:**
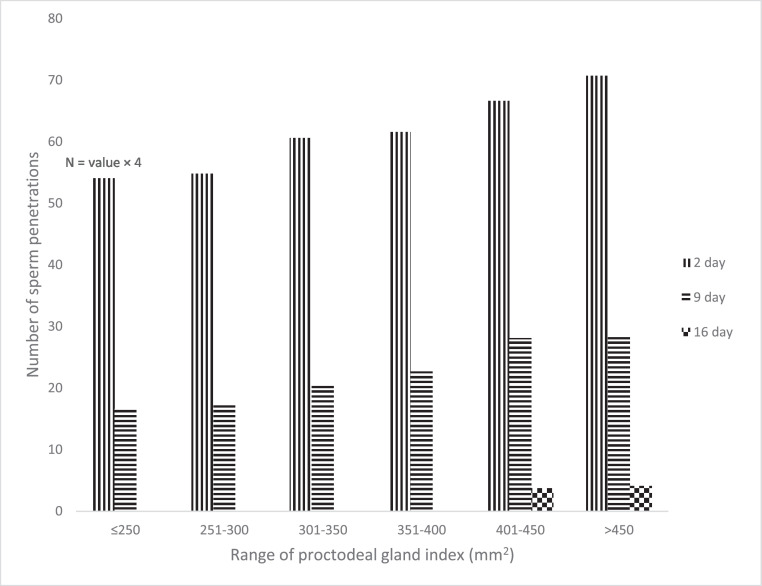
Number of sperm penetrated into the perivitelline membrane (area = 9 mm^2^) overlying the germinal disc in the different groups on the 2nd, 9th, and 16th d postinsemination.*The number of sperm penetrations on day 2 = the value multiplied by 4.

Figure 2 and Table 4 show the number and the mean rank of sperm penetrated into the perivitelline membrane overlying the germinal disc in the different groups on the 2nd, 9th, and 16th d postinsemination.

**Table 4:** Mean rank of sperm number penetrated into the perivitelline membrane (area = 9 mm^2^) overlying the germinal disc in the different groups on the 2nd, 9th, and 16th d postinsemination using Kruskall-Walist test.

Day postinsemination	Range of proctodeal gland index (mm^2^)						Kruskal-wallis test (chi-square)	Asymptotic. significance.
	1st G	2nd G	3rd G	4th G	5th G	6th G		
	(≤250)	(251-300)	(301-350)	(351-400)	(401-450)	(>450)		
2	3.5^e^	9.5^d^	16.7^c^	20.2^c^	27.5^b^	33.5^a^	33.442	<.0001
9	4.2^e^	8.8^e^	15.5^d^	21.5^c^	27.6^b^	33.4^a^	33.742	<.0001
16	12.5^b^	12.5^b^	12.5^b^	12.5^b^	29.4^a^	31.6^a^	33.559	<.0001

a–d Means with at least one common superscript in a row do not differ significantly (*p* > 0.05).

The Shapiro-Wilk goodness of fit test revealed non-normally distributed values for the number of sperms penetrating the perivitelline membrane. Consequently, the data pertaining to this variable were subjected to analysis using the Kruskal-Wallis test. Subsequent Dunns pairwise multiple comparison tests demonstrated a significant disparity in mean ranks of perforations in the perivitelline membrane overlaying the germinal disc. Specifically, a markedly higher significance was observed in the large-sized gland groups compared to both small and average-sized proctodeal gland groups on the 2nd and 9th d postinsemination. On the 16th d postinsemination, we could not observe any holes in the perivitelline membrane in small and average-size gland groups in contrast to that observed in large-sized gland groups.

A high significant (*P* < 0.0001) positive correlation between proctodeal gland index and testes weight (*R^2^* = 0.86), foam weight (*R^2^* = 0.99), testosterone level (*R^2^* = 0.97), ejaculate volume (*R^2^* = 0.97) and fertility (*R^2^* = 0.95) were observed in Japanese quail (Table 5).

**Table 5:** Pearson's correlation coefficients among the various measurement indices.

	FW (mg/bird)	Sexual libido time (s)	Testes weight (g)	Fertility (%)	Testosterone (ng mL-1)	Semen ejaculate volume (μL)	Frequency of foam discharge (24 h)
	D	F	M	Y	AC	AD	
Proctodeal gland index (mm^2^)	0.99^⁎⁎^	−0.95^⁎⁎^	0.98^⁎⁎^	0.95^⁎⁎^	0.97^⁎⁎^	0.97^⁎⁎^	0.97^⁎⁎^

FW (mg/bird) = Foam weight *Significant at 5% level, **significant at 1% level, NS = non-significant.

In addition, significant (*P* < 0.0001) positive correlations were observed between proctodeal gland index and TMOT%, PR%, VSL, VCL, VAP, LIN, WOB, and STR (Table 6). In contrast, a significant negative correlation was found with mean libido time, while no significant correlations were observed for MAD, ALH, and BCF.

**Table 6:** Pearson's correlation coefficients among proctodeal gland index, sperm motility, and kinematics in Japanese quail.

Variables	TMOT	PR	VSL	VCL	VAP	LIN	WOB	STR	MAD	ALH	BCF
Proctodeal gland index (mm^2^)	0.96^⁎⁎^	0.97^⁎⁎^	0.98**	0.86^NS^	0.81^NS^	0.98^⁎⁎^	0.97^⁎⁎^	0.97^⁎⁎^	-0.73^NS^	-0.66^NS^	-0.87^NS^

Abbreviations: TMOT, sperm total motility percentage; PR, progressive motility (%); VSL, velocity straight line; VCL, velocity curvilinear; VAP, velocity average path; LIN, linearity; WOB, wobble movement coefficient; STR, straightness; MAD, mean angular degree; ALH, lateral head displacement; BCF, beat-cross frequency. *Significant at 5% level; **significant at 1% level; NS, nonsignificant.

## DISCUSSION

In regard to reproductive strategies in birds, the male strategies involve rapid sperm production, maturation, and transport due to the limited storage capacity in their genital ducts, which can hold sperm for only 1 d (Clulow and Jones, 1982). After copulation, sperm cross the vagina, reach the uterovaginal junction, and are stored within the sperm storage tubules for a period that varies among species (from 2 to 10 wk). For quails, the average fertility period ranges from 6 to 8 days, which can be extended for up to 11 d (Sittmann and Abplanalp, 1965). This feature fulfills the female birds' reproductive strategy in that it allows the fertilization process to continue for a long time without the need for several repeated copulations when there are no males around. Also, if copulation occurs a few hours before egg release, it ensures that the sperm remain intact inside the oviduct and are not expelled.

In the current study, we found that proctodeal gland size in males significantly influences the fertility period in Japanese quail, as females copulated with small and average-sized proctodeal gland males produced fertile eggs for shorter periods than those mated with males having large-sized glands. Furthermore, the fertility rates in large-sized proctodeal gland groups were higher than those of small and medium-sized gland groups. Some theories were proposed to explain the prolonged duration of sperm residence in sperm storage tubules in birds. Froman (2003) suggested that spermatozoa maintain their residence in SST lumens by oscillatory, non-stoppable movement against the direction of a fluid flow (rheotaxis) generated through membrane integral proteins (aquaporins) located on epithelial cells. This movement allows sperm to swim at a velocity greater than or equal to that of the flow that maintains sperm position inside the SST; and when ATP reserves are depleted, sperm motility decreases, which results in sperm being swept out of the SST's lumen to the uterovaginal junction. The role of sperm rheotaxis in sperm selection through the vagina and sperm uptake and storage in the SSTs has been discussed in detail (see El-sherry et al., 2022; Sayed et al., 2023). This may indicate that the energy reserves in the mitochondria of sperm in males with a large proctodeal gland are greater than those in sperm of smaller-gland males. It is unfortunate that we did not estimate the size of mitochondria in sperm or the ATP content of sperm samples in this experiment. However, we measured sperm velocity using CASA. The results showed that the proportion of motile sperm and sperm kinematic parameters, i.e., VSL, VCL, and VAP values, were all significantly increased in association with increasing the proctodeal gland size. It is well documented that, in freshly collected spermatozoa, both the percentage of motile sperm and sperm swimming velocity showed a strong association with sperm ATP across species (Tourmente et al., 2013). Singh et al. (2012) artificially inseminated quails with an equal number of spermatozoa diluted in foam extract and normal saline and found that mixing sperm with foam resulted in higher fertility and a longer fertility period than sperm diluted in saline. The researchers concluded that proctodeal foam facilitates sperm transport into the oviduct by increasing sperm motility. However, in natural mating, although in different amounts, foam is secreted and mixed with spermatozoa from both large and small-sized proctodeal glands. This might indicate that foam concentration influences sperm motility and fertility rates. Biswas et al. (2010) reported that foam extract (1:1 weight to volume) diluted with saline (1:20 to give 5% foam extract) significantly increased sperm motility. However, concentrations of 10% and above suppressed sperm motility. These results increase the complexity of the assumption that foam accelerates sperm entry to oviducts by increasing sperm motility.

Contrary to Forman's hypothesis, others suggested that sperm typically acquire head-to-head agglutination (Van Krey et al., 1981) and remain in a quiescent state within the SSTs in birds such as chickens (Van Krey et al., 1981; Froman and Engel, 1989), quail (Bakst et al., 1994), and turkeys (Bakst and Bauchan, 2015). There is a possibility that the prolonged storage of sperm is due to the agglutinated mode of sperm in the SSTs (Bakst et al., 1994; Ito et al., 2011). According to Van Krey et al. (1981) random detachment of agglutinated sperm causes the gradual egress of spermatozoa into the oviduct lumen. In this case, the number of agglutinated sperm in the SSTs will be the determinant factor responsible for prolonging fertility.

The sperm fertilizing capacity was determined in this study using sperm-egg penetration assay which allowed us to measure the number of sperms that pass through the oviduct and initiate the first stage of fertilization by hydrolyzing the ova perivitelline layer. It was found that perivitelline membranes overlying germinal discs had significantly fewer holes on the 2nd and 9th days post-insemination in small and average-sized proctodeal gland groups than in big-sized gland groups. It is well established that fertility is strongly correlated with the number of holes and the sperm trapped over the germinal disc (Bramwell et al., 1995; Santos et al., 2013). In addition, males with large proctodeal glands had higher sperm concentrations in their ejaculates which might affect the number of sperms stored in the SSTs and consequently the number of sperm penetrations. The acrosomes of quail sperm have a high protease content or trypsin-like enzyme activity (Win et al., 2006). This enzyme is essential in digestion and for the optimum penetration into the vitelline membrane of the ova (a single layer of connective tissue-like protein) at the time of fertilization (Win et al., 2006). The proteolytic activity is directly influenced by the level of testosterone, which is positively correlated with the proctodeal gland index. Thus, birds that have a larger PAREA may have greater concentrations of trypsin-like enzymes or proteases in their sperm, which facilitates perivitelline membrane penetration.

Sperm velocity and progressive motility are extremely important and must be evaluated together when evaluating sperm quality because they determine the success of the sperm in passing through the oviduct and its ability to reach the fertilization site (Sayed et al., 2017). In the current study, it was found that males with larger proctodeal glands had higher values of sperm motility (*p* < 0.05) and velocity parameters compared to those with small-sized glands. The proctodeal gland size and foam production thus appear to be potent effectors of motility for ejaculated Japanese quail spermatozoa (Shit et al., 2010). Slow-speed progressive sperm are unlikely competitive because they most likely fail to cross the vagina, ascend the oviduct and do not reach the funnel. Likewise, sperm with rapid nonprogressive motility swim in small circles and do not move forward; therefore they also fail to reach the ova at the fertilization site (Sloter et al., 2006). Hence, the efficiency of fertilization depends primarily on the presence of a high proportion of sperm with rapid progressive motility and higher VSL values (Sayed et al., 2017). Many studies have correlated sperm kinematics, particularly sperm velocity (VCL, VSL, and VAP), with their fertilizing capacity and fertility outcomes. For example, in bulls, sperm kinematic parameters VCL, VSL, and VAP in frozen/thawed sperm were correlated with achieving pregnancy (Nagy et al., 2015). Out of the 3 kinematic parameters, VAP shows the highest correlation with fertility and may be the most useful sperm velocity parameter for estimating sperm fertility (Nagy et al., 2015). This indicates that males with larger proctodeal glands have better sperm quality and higher fertilizing potential. Also, the amplitude of lateral head displacement (ALH) along with 3 kinematic values (VCL, VSL, and VAP) were reported as predictors of IVF outcome (Hirano et al., 2001; Shibahara et al., 2004). Moreover, VCL, ALH, and linearity (LIN) are reported to be associated with sperm hyperactivated motility, which enables sperm to traverse the fallopian tube and fertilize the oocyte (Mortimer, 2000). An inverse relationship between sperm velocity parameters and the degree of sperm DNA fragmentation was reported. Sperm cells with higher VAP, VCL, and VSL have a lower sperm DNA fragmentation rate and higher fertility potential (Hirano et al., 2001; Shibahara et al., 2004). Also, a significant relationship was found between the linearity of the average path (STR), the curvilinear path intersection (BCF) and the fragmentation of sperm DNA (Aghazarian et al., 2021).

The development and function of the proctodeal gland and foam production are primarily dependent on androgen and are positively related to the size and activity of the testes, sperm production, plasma testosterone level, and fertility in sexually active male quail (Mohan et al., 2002; Balthazart et al., 2003; Biswas et al., 2007 a,b and Singh et al., 2011b). Studies have indicated the presence of androgen receptors in the proctodeal glandular cells of quail and that the development and secretory activity of the proctodeal gland are promoted by androgen in a receptor-mediated manner (Kaku et al., 1993). Therefore, males with large PAREA produce more foam and semen, secrete higher testosterone levels, and are therefore more fertile compared with males with smaller PAREA.

## CONCLUSIONS

It can be concluded that males with large proctodeal glands are more fecund, as they have larger testes, higher circulating testosterone concentrations, produce more sperm with higher motility and velocity, higher sperm longevity as indicated by prolonged fertility duration in females, and higher sperm fertilizing capacity as indicated by a higher number of sperm penetrations into the perivitelline membrane, thereby having higher rates of fertility. Consequently, maintaining males with a large proctodeal gland index in a breeding program allows for better fertility and may help reduce the number of males needed to maintain optimal fertility.
